# Caregiving Together: The Relationship Between Paid and Family Caregivers in the Home

**DOI:** 10.1093/geroni/igaa057.1894

**Published:** 2020-12-16

**Authors:** Jennifer Reckrey, Robyn Stone

## Abstract

Family caregivers provide the lion’s share of care that allows older adults with functional impairment to remain living at home. Yet as care needs grow, many older adults and their families turn to paid caregivers (e.g. home health aides, personal care attendants, and other direct care workers) to provide additional support. While evidence suggests that paid and family caregivers work together to provide increasingly complex care at home, research that describes this important collaboration is limited. In this symposium, we present innovative and interdisciplinary research that highlights the overlap between family caregiving and long-term care workforce research. We begin by presenting two studies that focus on populations where paid caregivers may have outsized impact on family caregivers: Reckrey et al report that receipt of 20+ hours of paid caregiving per week was associated with less caregiver strain among family caregivers of those with advanced dementia and Falzarano et al report that home care hours mediated the association between caregiver stressors and negative effects of caregiving among long-distance family caregivers. Franzosa et al then describe home health aides’ perceptions of relationship dynamics as aides and family members negotiate care tasks in the home. Finally, Gallopyn et al explore scenarios where paid and family caregiver roles blur (e.g., family caregivers receiving payment for providing care, paid caregivers with extensive experience as family caregivers). Taken together, these studies describe critical ways paid and family caregiver experiences are intertwined and highlight the importance of ongoing research about this collaboration.

